# Microenvironment-dependent growth of Sezary cells in humanized IL-15 mice

**DOI:** 10.1242/dmm.050190

**Published:** 2023-10-13

**Authors:** Jie Gao, Shumei Ren, Gabrielle Choonoo, Guoying Chen, Davor Frleta, Jun Zhong, Namita Gupta, Prachi Sharma, Adelekan Oyejide, Gurinder S. Atwal, Lynn Macdonald, Andrew Murphy, Frank Kuhnert

**Affiliations:** Regeneron Pharmaceuticals, Inc., Tarrytown, NY 10591, USA

**Keywords:** Sezary syndrome, CTCL, IL-15, PDX, Humanized mice

## Abstract

Sezary syndrome (SS) is a rare, aggressive leukemic variant of cutaneous T-cell lymphoma (CTCL) that lacks adequate therapeutic options and representative small-animal models. Here, we demonstrate that IL-15 is a critical CTCL growth factor. Importantly, an immunodeficient knock-in mouse model genetically engineered to express human IL-15 uniquely supported the growth of SS patient samples relative to conventional immunodeficient mouse strains. SS patient-derived xenograft (PDX) models recapacitated key pathological features of the human disease, including skin infiltration and spread of leukemic cells to the periphery, and maintained the dependence on human IL-15 upon serial *in vivo* passaging. Detailed molecular characterization of the engrafted cells by single-cell transcriptomic analysis revealed congruent neoplastic gene expression signatures but distinct clonal engraftment patterns. Overall, we document an important dependence of Sezary cell survival and proliferation on IL-15 signaling and the utility of immunodeficient humanized IL-15 mice as hosts for SS – and potentially other T and NK cell-derived hematologic malignancies – PDX model generation. Furthermore, these studies advocate the thorough molecular understanding of the resultant PDX models to maximize their translational impact.

## Teaser

**Summary:** The genetic humanization of IL-15 in immunodeficient host mice uniquely supports the engraftment and growth of Sezary syndrome (SS) patient samples, resulting in the generation of new SS patient-derived xenograft models.

## INTRODUCTION

Cutaneous T-cell lymphoma (CTCL) is a heterogeneous group of non-Hodgkin lymphomas derived from malignant transformation of T cells that home to and populate the skin (Brunner et al., 2020; Dummer et al., 2021; Phyo et al., 2020). The most frequent form of CTCL is mycosis fungoides (MF), where tumor cell localization is restricted to skin. Sezary syndrome (SS) is a rare and aggressive leukemic variant of CTCL characterized by erythroderma, lymphadenopathy and atypical lymphocytes (Sezary cells) circulating in peripheral blood (Kubica et al., 2012). Circulating Sezary cells exhibit an abnormal immunophenotype: an expanded CD4^+^ T-cell population resulting in a CD4/CD8 ratio of more than 10 as well as loss of CD7 and CD26 (also known as DPP4) (Dummer et al., 2021; Kubica et al., 2012; Willemze et al., 2005). The pathogenic mechanisms of CTCL are not entirely understood, and no single cancer-initiating factor has been identified. Primary CTCL cells grow poorly *in vitro* even in the presence of multiple cytokines, growth factors and stromal cells (Berger et al., 2002; Yamanaka et al., 2006), indicating that signals provided by the tumor microenvironment are critical to support their survival and growth. Few MF and SS cell lines are available, and subcutaneous cell line-based tumor models do not represent the clinical features of MF/SS (Krejsgaard et al., 2010; Thaler et al., 2004). A small number of SS patient samples have been directly engrafted into immunodeficient mice (Poglio et al., 2021; Townsend et al., 2016; Wu et al., 2021). However, the disease take rate is typically low (Poglio et al., 2021), and these models do not exhibit spread of Sezary cells to peripheral blood and lymphoid tissues (Townsend et al., 2016; Wu et al., 2021). Overall, better and more representative preclinical SS models are needed to study disease biology and for preclinical therapeutic testing.

Interleukin (IL)-15 is a pleiotropic cytokine important for the development, proliferation, survival and activation of natural killer (NK), T and B cells (Mishra et al., 2014; Waldmann et al., 2020). The heterotrimeric IL-15 receptor is composed of the common gamma chain (γc) subunit (encoded by *IL2RG*; also known as *CD132*), the β chain subunit IL-2/15Rβ (encoded by *IL15RB*; also known as *CD122*) shared with the IL-2 receptor, and a private IL-15 specific α subunit IL-15Rα (encoded by *IL15RA*). IL-15Rα is a high-affinity receptor for IL-15. IL-15 binds to either the membrane-bound or the soluble form of IL-15Rα and is then presented *in trans* to the IL-15Rβγ complex expressed on nearby effector cells to initiate cellular signaling. IL-15 downstream signaling pathways include the JAK/STAT, PI3K/AKT and MAPK pathways, with resultant transcriptional activation of the proto-oncogenes c-Fos (encoded by *FOS*), c-Jun (encoded by *JUN*) and c-Myc (encoded by *MYC*), and the antiapoptotic protein BCL-2 (Mishra et al., 2014; Waldmann et al., 2020). Recent data have implicated IL-15 signaling in the pathogenesis of CTCL. Skin lesions of CTCL patients show overexpression of IL-15 protein (Leroy et al., 2001), and IL-15 has been implicated in CTCL cell growth and survival *in vitro* (Döbbeling et al., 1998; Marzec et al., 2008). Furthermore, ubiquitous transgenic overexpression of IL-15 in mice resulted in the development of CTCL (Mishra et al., 2014). Overall, published data support both microenvironmental (paracrine) and autocrine IL-15 signaling mechanisms in driving CTCL development.

Mouse IL-15 does not bind to and activate the human IL-15 receptor. Here, we demonstrate that the genetic humanization of IL-15 in immunodeficient mice to overcome the lack of cross-species reactivity dramatically improved SS patient sample take rate and disease burden. Established SS patient-derived xenograft (PDX) models exhibited key clinical features of the human disease and maintained their dependence on human IL-15 upon serial *in vivo* passaging.

## RESULTS

### IL-15 promotes Hut78 CTCL cell proliferation *in vitro*

**Fig. 1.**
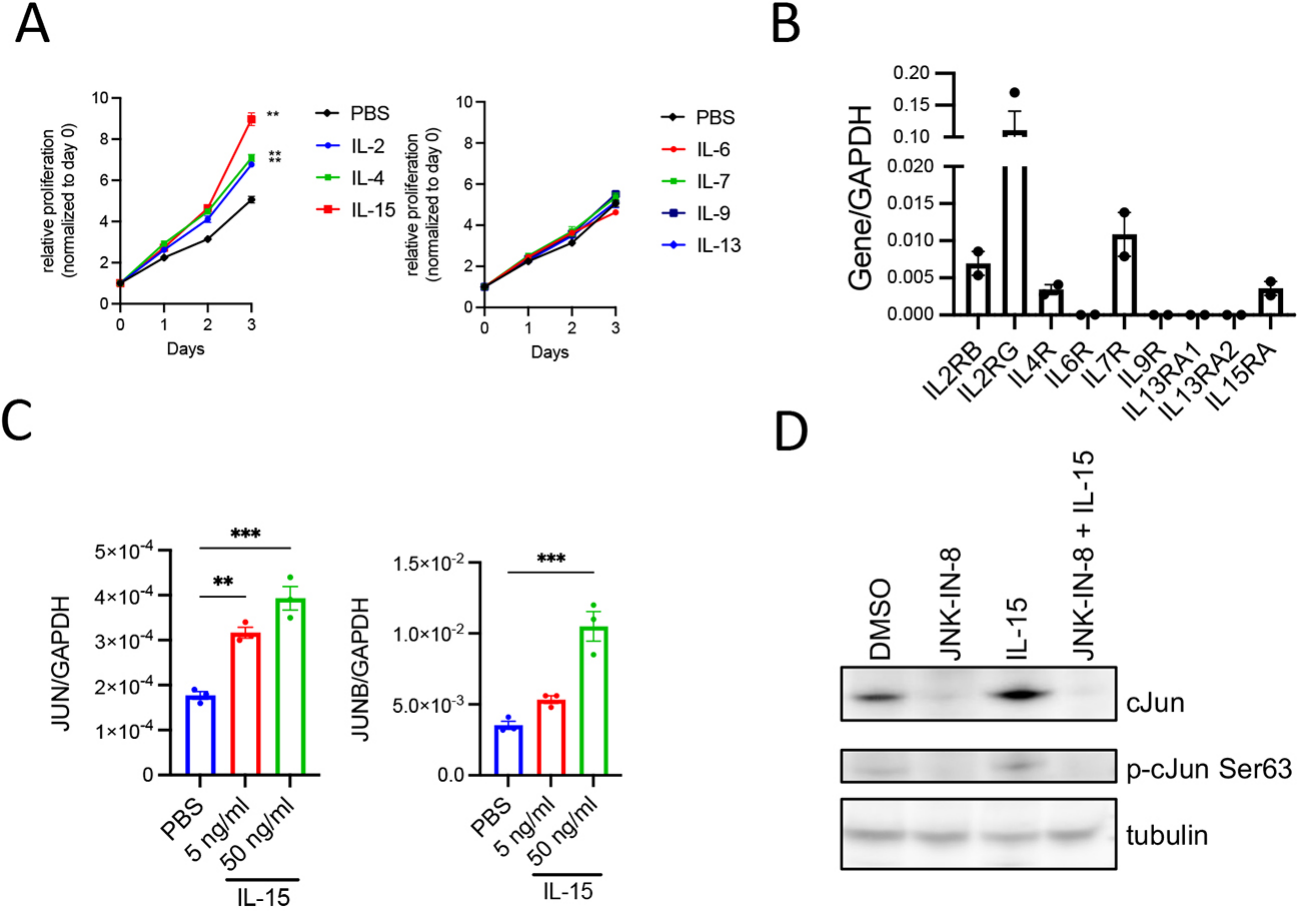
**IL-15 promotes cutaneous T-cell lymphoma (CTCL) cell proliferation *in vitro*.** (A) Proliferation of Hut78 cells treated with different cytokines at 50 ng/ml. Each condition was tested in technical triplicate. Data represent mean±s.e.m. Two-way ANOVA with Dunnett's multiple comparisons test, comparing with PBS at day 3. **P<0.01. (B) Expression of cytokine receptors in Hut78 cells by quantitative PCR (qPCR). qPCR assays were set up in technical duplicates. Data represent mean±s.e.m. (C) Analysis of *JUN* and *JUNB* gene expression by qPCR in Hut78 cells treated with IL-15 for 24 h. Gene expression was normalized to *GAPDH*. qPCR assays were set up in technical triplicates. Data represent mean±s.e.m. Ordinary one-way ANOVA with Dunnett's multiple comparisons test, comparing with PBS. **P<0.01, ***P<0.001. (D) Western blot analysis of Hut78 cells treated with 2 μM JNK-IN-8, 50 ng/ml IL-15 or their combination for 24 h. Representative results from one of two independent experiments are shown.

To test which T-cell cytokines have growth-promoting effects on CTCL cells, we screened a panel of cytokines in Hut78 cells, a cytokine-independent CTCL cell line, for proliferation induction activity. The cytokines tested included IL-2, IL-7 and IL-15, growth factors essential for lymphocyte function, the Th2 cytokines IL-4 and IL-13, reported to have increased serum levels in SS patients (Geskin et al., 2015), and IL-6 and IL-9, which have been implicated in CTCL pathogenesis (Olszewska et al., 2021; Vieyra-Garcia et al., 2016). Consistent with results in other CTCL cell lines (Döbbeling et al., 1998; Marzec et al., 2008), IL-2, IL-4 and IL-15 significantly increased Hut78 *in vitro* cell proliferation, with IL-15 appearing most potent (1.8-fold increase). IL-6, IL-7, IL-9 and IL-13 had no impact on Hut78 cell proliferation (Fig. 1A). Taqman analysis confirmed expression of the relevant receptor subunit-encoding genes *IL2RB*, *IL2RG*, *IL4R* and *IL15RA* in Hut78 cells (Fig. 1B). IL-15 treatment increased *JUN* and *JUNB* target gene expression in a dose-dependent manner in Hut78 cells (Fig. 1C). Similarly, IL-15 increased protein expression and activation of c-Jun (Fig. 1D). Treatment with a JNK inhibitor, JNK-IN-8, blunted the upregulation and activation of c-Jun, implicating JNK signaling as a mediator of c-Jun target gene expression downstream of the IL-15 signaling pathway (Fig. 1D). IL-15 is produced by several cell types and tissues, including skin (Adachi et al., 2015). To assess IL-15 levels in clinical CTCL samples, immunohistochemistry for IL-15 was performed on skin tissues of CTCL patients and unaffected controls. This analysis demonstrated IL-15 upregulation in skin from CTCL patients relative to skin from controls (Fig. S1). Overall, these results are consistent with a growth-promoting function of IL-15 in CTCL.

### Humanization of IL-15 in immunodeficient mice supports Sezary cell growth *in vivo*

To assess whether IL-15 promotes CTCL growth *in vivo*, we genetically engineered immunodeficient SRG mice (humanized for *SIRPA*, on a *Rag2^−/−^Il2rg^−/−^* background) to express human IL-15 in place of its murine counterpart (Herndler-Brandstetter et al., 2017) (referred to as SRG15 mice hereafter). Human *IL15* expression and the concomitant loss of mouse *Il15* in tissues, including skin, from SRG15 mice were confirmed by Taqman analysis (Fig. S2A; Herndler-Brandstetter et al., 2017). Human IL-15 can bind mouse IL-15Rα, which in turn trans-presents it to the human IL-2/IL-15Rβγ complex (Fig. S2B; Herndler-Brandstetter et al., 2017). Initially, we compared growth of Hut78 cells in parental SRG versus SRG15 mice. Consistent with the *in vitro* results, human IL-15 significantly increased the growth of subcutaneous Hut78 tumors *in vivo* (Fig. S2C).

**Fig. 2.**
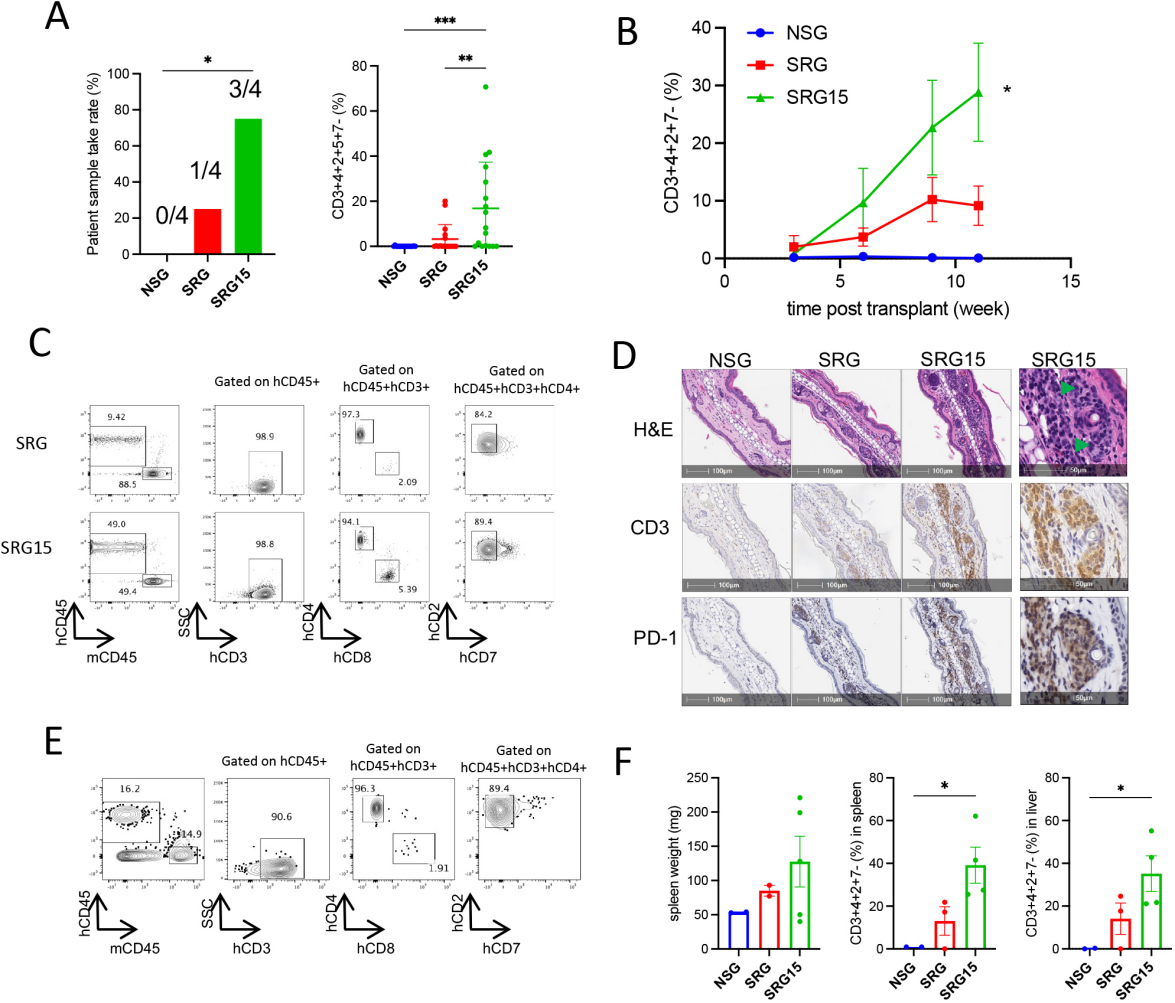
**Sezary syndrome (SS) patient-derived xenograft (PDX) model generation in SRG15 mice.** (A) SS take rate (right) and disease burden (left) in peripheral blood 8 weeks post xenograft in NSG, SRG and SRG15 mice. A total of four patient samples were xenografted. Each patient sample was xenografted into three to five mice per strain. Each dot in the right panel represents one individual mouse combined for all four PDX samples. Statistical significance of take rate (left) was determined by Chi-square test, *P<0.05. Data in right panel represent mean±s.e.m. Ordinary one-way ANOVA with Turkey's multiple comparisons test. **P<0.01, ***P<0.001. (B) Patient sample 1 was xenografted into NSG, SRG and SRG15 mice (*n*=5 per group). Disease burden in peripheral blood as measured by frequency of mCD45^−^hCD45^+^hCD3^+^hCD4^+^hCD2^+^hCD7^−^ cells over time was plotted. Data represent mean ±s.e.m. Two-way ANOVA with Dunnett's multiple comparisons test, comparing SRG15 and NSG mice at week 12. *P<0.05. (C) Representative flow cytometry plots of peripheral blood from SRG and SRG15 mice at 9 weeks post xenograft. (D) H&E staining, and CD3 and PD-1 immunohistochemistry of ear punch specimens at 9 weeks post xenograft. Green arrowheads in 100× high-magnification image on the right indicate Sezary cells with cerebriform nuclei in a SRG15 mouse. (E) Immunophenotype of cells isolated from the skin of SRG15 mouse at 12 weeks post xenograft. (F) Spleen weight (left) and disease burden in spleen (middle) and liver (right) at 12 weeks post xenograft. Data represent mean±s.e.m. Ordinary one-way ANOVA with Dunnett's multiple comparisons test. *P<0.05.

Next, we tested the effects of human IL-15 on growth and take rate of SS patient samples. Four SS patient peripheral blood samples (Table S1) were intravenously engrafted into sublethally irradiated conventional immunodeficient NSG, parental SRG and engineered SRG15 mice. Host mice were irradiated to ablate myeloid cells to enhance SS cell engraftment. Disease burden in mice was monitored by flow cytometry analysis of peripheral blood for mCD45^−^hCD45^+^hCD3^+^hCD4^+^hCD2^+^hCD7^−^ leukemic cells. Peripheral blood leukemic cell burden of greater than 0.5% of all mononuclear cells was defined as successfully engrafted. None of the SS patient samples grew in the NSG mice, whereas three of four SS patient samples engrafted in the SRG15 mice. One of four samples engrafted in the parental SRG mice (Fig. 2A, left). Leukemic disease burden in individual animals at 8 weeks post transplant for the SS patient sample that engrafted was significantly higher in SRG15 than in SRG mice, with faster kinetics of disease development (Fig. 2A, right, B; Fig. S3A). Engrafted Sezary cells in SRG and SRG15 mice were dominated by a CD4^+^ immunophenotype with lack of CD7 expression, consistent with the clinical features of SS patient samples (Fig. 2C). Abundant CD3^+^ lymphocyte infiltration into skin was detected in SRG15 mice (Fig. 2D; Fig. S3B). Skin-infiltrating cells exhibited characteristic cerebriform nuclei with condensed chromatin and stained positive for PD-1 (also known as PDCD1), key features of SS cells (Fig. 2D). Consistent with the analysis of the original patient sample, the immunophenotype of cells isolated from skin of SRG15 mice was hCD3^+^hCD4^+^hCD8^−^hCD2^+^hCD7^−^ (Fig. 2E). Additionally, leukemic cell infiltration into liver and spleen and associated splenomegaly was observed in SRG15 mice (Fig. 2F).

### Established SS PDXs can be serially passaged in SRG15 mice

**Fig. 3.**
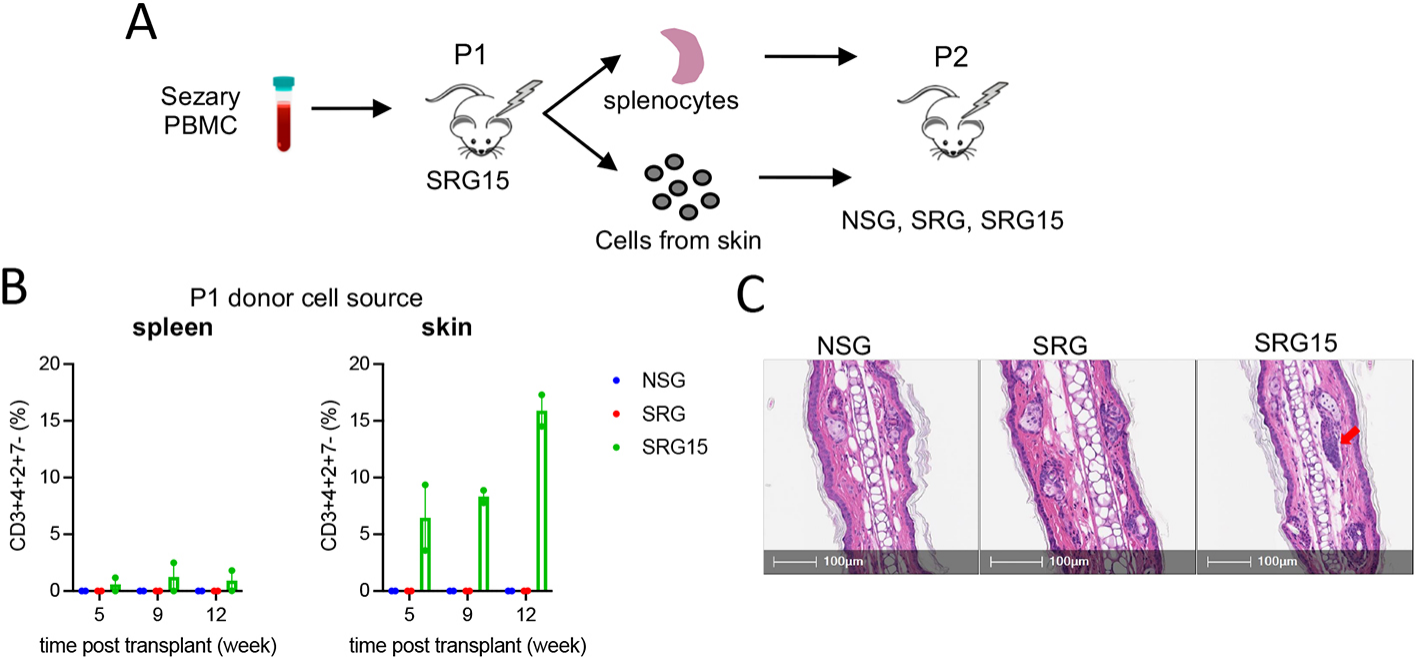
**Serial passaging of SS PDX in SRG15 mice.** (A) Schematic of secondary passage of SS PDX. Cells from the spleen or skin of postnatal day (P)1 mice were isolated and intravenously injected into secondary recipient NSG, SRG or SRG15 mice. (B) Disease burden in peripheral blood over time in P2 mice as measured by the frequency of mCD45^−^hCD45^+^hCD3^+^hCD4^+^hCD2^+^hCD7^−^ cells. Left panel refers to donor cells isolated from P1 spleen; right panel refers to donor cells isolated from P1 skin. Data represent mean±s.e.m. (C) H&E staining of ear punch specimens at 9 weeks post secondary xenograft. The red arrow indicates a lymphocyte cluster in an SRG15 mouse. PBMC, peripheral blood mononuclear cell.

Next, we addressed whether SS PDX models established in SRG15 mice could be serially *in vivo* passaged. Freshly isolated total cells from spleen and skin of postnatal day (P)1 SRG15 mice with high engraftment levels (>15% in peripheral blood) were intravenously transplanted into irradiated P2 NSG, SRG or SRG15 mice (Fig. 3A). Circulating Sezary cells were readily detected in secondary SRG15 hosts, but not in NSG or SRG mice (Fig. 3B), indicating the continued dependence on microenvironmental IL-15 to support leukemic cell growth and survival *in vivo*. Skin-derived cells from P1 SRG15 mice exhibited increased engraftment and overall increased disease burden in secondary SRG15 hosts compared to spleen-derived cells from the same mice (Fig. 3B). Similar to primary SRG15 hosts, lymphocyte infiltration into skin was detected in P2 SRG15 hosts but not in age-matched SRG or NSG mice (Fig. 3C). Collectively, these data demonstrate that SRG15 mice are preferred hosts for SS patient samples relative to NSG or SRG mice, affording increased take rate and disease burden, as well as faithful recapitulation of pathological disease features.

### Skin-resident Sezary cells possess malignant gene expression signature

**Fig. 4.**
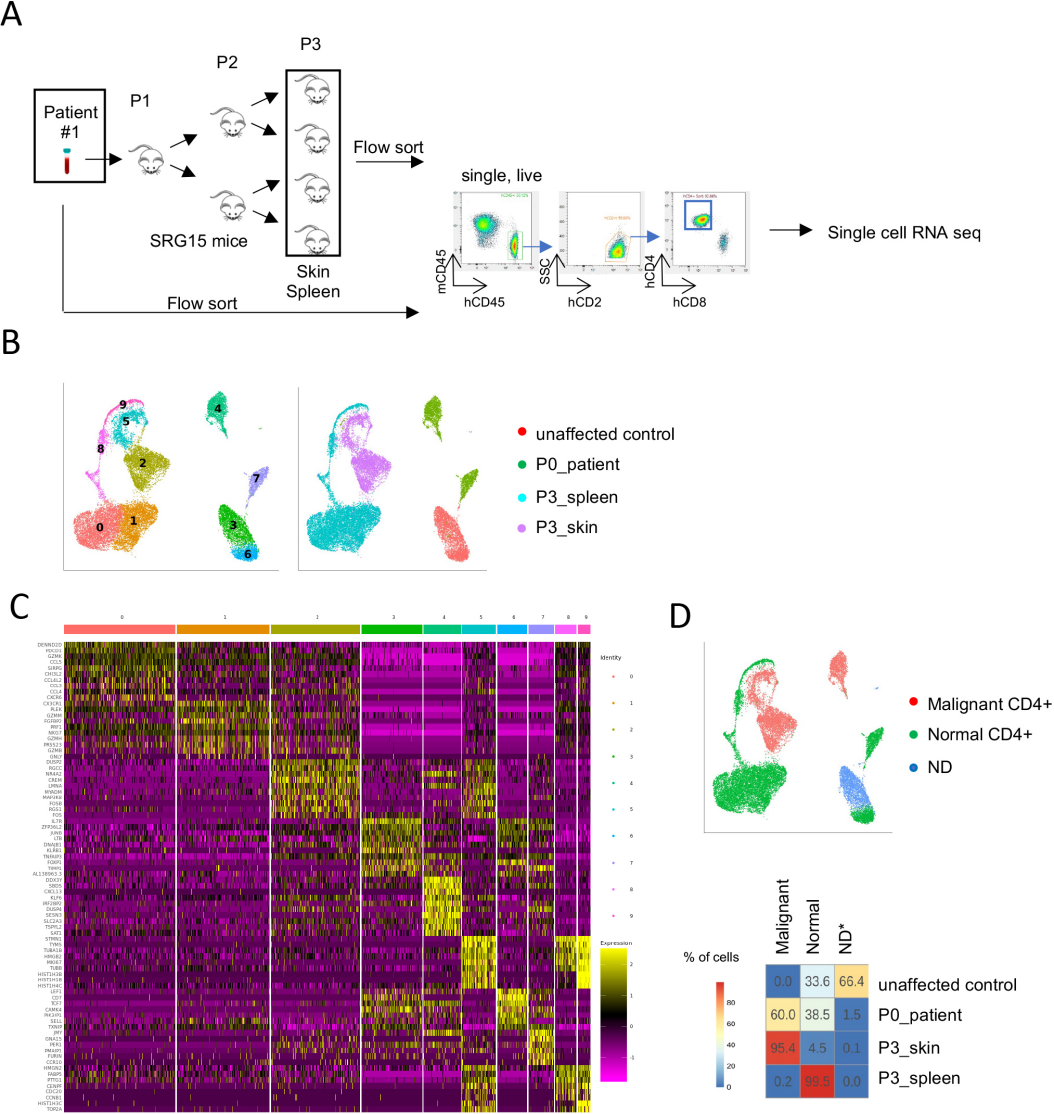
**Single-cell analysis revealed that skin-resident Sezary cells exhibit malignant gene expression signature.** (A) Sample preparation workflow for downstream single-cell RNA-sequencing analysis. Skin and spleen samples from four P3 SS PDX mice as well as PBMCs from the original SS patient sample and an unaffected donor (control) sample were collected. Cells were sorted using flow cytometry for CD4^+^ cells (mCD45^−^hCD45^+^hCD2+hCD4^+^hCD8^−^). (B) Uniform manifold approximation and projections (UMAPs) of 22,745 CD4^+^ cells from control PBMCs, SS patient PBMCs, and P3 mouse skin and spleen samples. The left UMAP is colored by cluster and the right UMAP is colored by sample group. (C) Heatmap of the top ten differentially expressed genes in each cluster. (D) Clusters were defined as normal, malignant or not determined (ND). The top UMAP is colored by cell type and the bottom heatmap shows the percentage of cell types for each sample.

To gain a better understanding of cellular composition and heterogeneity of the engrafted Sezary samples, we performed single-cell RNA-sequencing (scRNA-seq) analysis. We used flow cytometry to sort CD4^+^ cells (mCD45^−^hCD45^+^hCD2^+^hCD4^+^hCD8^−^) from the skin and spleen of four engrafted P3 SRG15 mice, the original SS patient sample and one unaffected donor (control) sample for this analysis (Fig. 4A). A total of 22,745 cells were used for downstream clustering analysis. Ten clusters were identified (Fig. 4B,C); the top differentially expressed genes in each cluster are listed in Table 1. Control donor CD4^+^ cells clustered separately from SS patient CD4^+^ cells (clusters 3 and 6 versus clusters 4 and 7), reflecting the transformed state of Sezary leukemic cells. Analysis of engrafted Sezary cells demonstrated that skin-resident CD4^+^ cells (clusters 2 and 5) clustered separately from the main spleen-resident CD4^+^ cell populations (clusters 0 and 1), suggesting tissue-specific regulation of gene expression programs. Genes highly expressed in clusters 2 and 4, including *DUSP2*, *NR4A2*, *LMNA*, *FOS*, *SLC2A3*, and *DUSP4*, were previously reported to be associated with Sezary leukemic cells (Borcherding et al., 2019; Moerman-Herzog et al., 2020). Genes highly expressed in cluster 5 (*STMN1*, *MKI67*) are associated with cell proliferation. Differential gene expression analysis of CD4^+^ T cells from the original SS patient sample and P3 skin and spleen from engrafted mice confirmed the tissue-specific regulation of Sezary cell gene transcription (Fig. S4, Table S2).

**Table 1.**
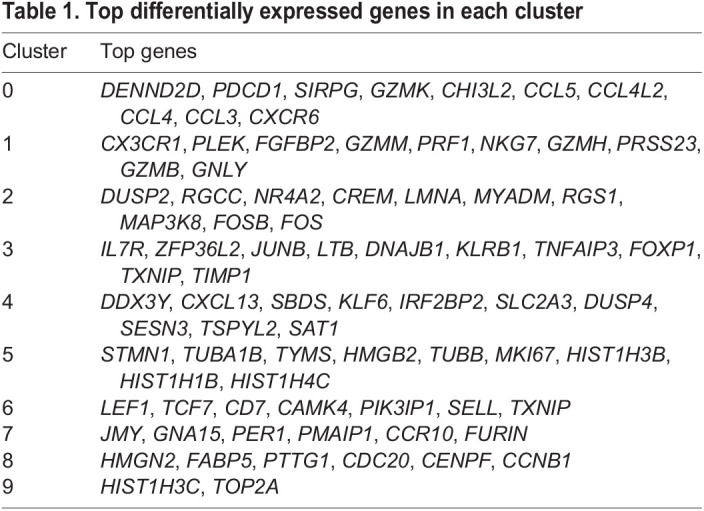
Top differentially expressed genes in each cluster

Next, we projected the normal versus malignant Sezary CD4^+^ cell gene expression signatures previously identified (Borcherding et al., 2019) onto our scRNA-seq dataset. Consistent with the clustering analysis, ∼60% of primary patient Sezary CD4^+^ cells scored as malignant while the remainder tracked as normal T cells, reflecting the heterogeneity of the patient's peripheral blood CD4^+^ compartment. Strikingly, almost all (95%) of the skin-resident CD4^+^ cells in the engrafted SRG15 mice exhibited the malignant gene signature, whereas the splenic CD4^+^ cells from the same animals scored quantitatively as normal (Fig. 4D; Table S3), a finding that suggests tissue-specific regulation of gene expression. To further characterize the SS PDX samples, we performed immunophenotyping analysis of CD4^+^ skin- and spleen-infiltrating cells from P2 and P3 SRG15 mice to assess the expression of canonical T-cell markers (CD5, CD7, TRBC1), activation markers (ICOS, PD-1) and CTCL markers (CCR4, KIR3DL2, EPHA4). This analysis revealed an overall stable immunophenotype for the markers examined between skin and spleen and across the different passages (Fig. S5). Among the makers analyzed, CD5, ICOS, PD-1, TRBC1 and KIR3Dl2 were expressed in the SS PDX samples. Therapeutic agents targeting these makers are currently in clinical development (Chavez et al., 2023; Khodadoust et al., 2020; Maciocia et al., 2017; Marie-Cardine et al., 2014), overall highlighting the utility of this model for preclinical therapeutic testing (Fig. S5).

In addition to the expression analysis, we performed single-cell T-cell receptor (TCR) sequencing on CD4^+^ cells to understand the clonal selection process during PDX engraftment. TCRs were defined by usage of TCR beta chain V gene, J gene and complementarity-determining region 3 (CDR3), and analyzed (Tables 2-4). The SS patient CD4^+^ cells were characterized by one predominant TCR clone (clone D) with a frequency of 60%, whereas, as expected, the TCR repertoire of the control was distributed evenly across many different TCR clones (Table 4). Skin and splenic CD4^+^ cells from the engrafted PDX samples were dominated by the same TCR clone (clone A), with the frequency ranging from 58% and 100% for individual P3 SRG15 mice (Table 3). Interestingly, this clone was not the dominant TCR clone in the original patient sample, suggesting strong selection for this subclone in the SRG15 mice.

**Table 2.**
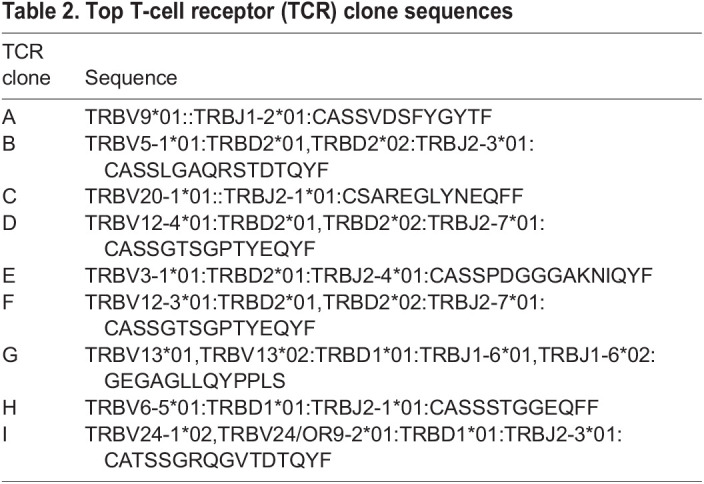
Top T-cell receptor (TCR) clone sequences

**Table 3.**
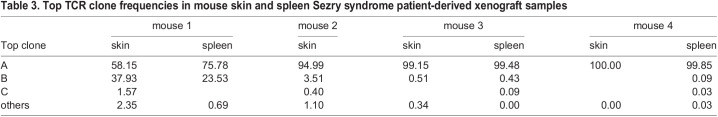
Top TCR clone frequencies in mouse skin and spleen Sezry syndrome patient-derived xenograft samples

**Table 4.**
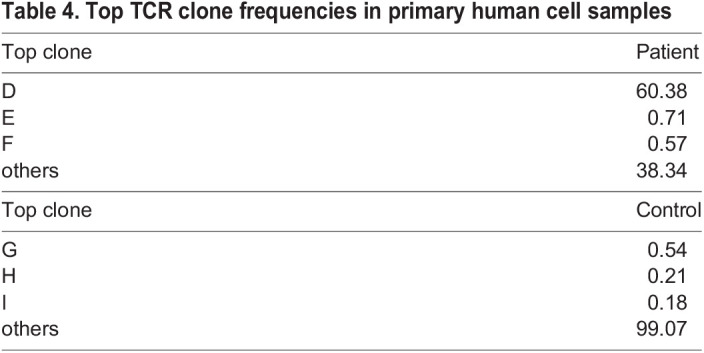
Top TCR clone frequencies in primary human cell samples

## DISCUSSION

The lack of mouse/human inter-species cross-reactivity of hematopoietic cytokines and growth factors constitutes a major obstacle to growing human hematopoietic cells in immunodeficient mice. One strategy to address this issue is the genetic humanization of the murine factors in the host mice. Preferably, this would be achieved by replacing the mouse gene with the human ortholog via gene knock-in approaches to (1) abrogate expression of the endogenous mouse gene and (2) ensure physiological expression levels of the introduced human gene. Genetic humanizations can be tailored to support engraftment and growth of specific hematopoietic lineages. Several studies have highlighted the overall viability of this approach, demonstrating, for instance, the enhanced survival of human red blood cells and the improved development and function of human NK cells in cytokine humanized immunodeficient mice (Herndler-Brandstetter et al., 2017; Song et al., 2021). This approach can also be applied to the generation of hematopoietic PDX tumor models, and has been shown to result in improved engraftment rates and faithful recapitulation of human disease features for acute myeloid leukemias and multiple myeloma patient samples, and even the generation of preneoplastic disease models (Das et al., 2016; Rongvaux et al., 2014).

Here, we build on this body of work and demonstrate that the genetic humanization of IL-15 uniquely supports the engraftment and growth of SS patient samples in immunodeficient mice. SS is a rare, aggressive leukemic variant of CTCL that lacks adequate therapeutic options and representative small-animal models. We started out by identifying IL-15 as a potent proliferation factor for the CTCL cell line Hut78, confirming previous results with other CTCL cell line models (Döbbeling et al., 1998; Marzec et al., 2008). This prompted us to test the utility of the humanized IL-15 SRG immunodeficient mouse strain (SRG15 mice) as hosts for SS patient samples. The SRG15 mice were generated via knock-in of the human *IL15* gene into the endogenous murine *Il15* locus and were previously shown to increase human NK cell growth and functionality (Herndler-Brandstetter et al., 2017). The humanization of IL-15 in SRG15 mice markedly increased SS sample take rate relative to that in parental SRG and conventional NSG immunodeficient mice. Notably, three of four SS samples engrafted in the SRG15 mice, whereas none of the patient samples grew in NSG mice. The fact that one of four SS samples engrafted in parental SRG mice seems to indicate that the mouse strain background (SRG versus NSG) is a contributing factor to patient sample engraftment frequency. Nevertheless, disease burden was significantly higher in SRG15 than that in SRG mice for the one patient sample that engrafted in the parental strain, highlighting the effect of human IL-15 on Sezary cell *in vivo* growth. Importantly, the SS PDX models generated in the SRG15 mice recapitulated the key pathological features of the human disease, including skin infiltration and spread of leukemic cells to the periphery. Although the overall number of engrafted SS patient samples in this study is on the low side (*n*=4), as SS is a rare disease and patient samples are difficult to procure, the differences in engraftment rates between the NSG and SRG15 strains are statistically significant, highlighting the benefits of this approach. Adoption of the IL-15 humanization platform by additional investigators should aid in further substantiating our findings.

The SRG15 approach reported here seems to afford several improvements in terms of experimental approach (e.g. intravenous implantation), take rate and/or disease manifestations over previously reported SS PDX modeling efforts. SS modeling studies by Poglio et al. (2021), van der Fits et al. (2012) and Wu et al. (2021) reported the generation of a limited number of SS PDX models that are encumbered by complex transplantation techniques (intrafemoral or intrahepatic engraftment), low engraftment efficiencies, and/or lack of skin or peripheral blood involvement. More recently, another immunodeficient strain expressing transgenic IL-15, NSG-IL-15, was reported (Abeynaike et al., 2023). Both NSG-IL-15 and SRG15 strains appear to exhibit physiological levels of human IL-15 expression, and human CD34^+^ constitution results in similar NK cell frequencies in peripheral blood. Based on these findings, one would expect that the NSG-IL-15 mice enhance SS cell engraftment similarly to the SRG15 mice.

Importantly, the SS PDX models generated in the SRG15 mice could be *in vivo* passaged and expanded *in vivo*, enabling the preclinical testing of therapeutic agents. Importantly, the established SS PDX model exhibited a stable immunophenotype and expressed several cell surface targets (ICOS, PD-1, TRBC1 and KIR3DL2) that are currently the subject of early clinical investigation in peripheral T-cell lymphoma (Chavez et al., 2023; Khodadoust et al., 2020; Maciocia et al., 2017; Marie-Cardine et al., 2014). The established SS PDX models maintained their dependence on human IL-15 as they could not be propagated in SRG or NSG mice. This strict dependence on human IL-15 raises the possibility that the IL-15 signaling axis could be a therapeutic target in CTCL. On the flip side, the fact that additional cytokines (IL-2, IL-4) induced proliferation in Hut78 cells suggests cytokine redundancy and possible resistance mechanism to IL-15 blockade. In the context of PDX modeling, it would be interesting to test whether the humanization of non-cross-reactive cytokines IL-4 and/or IL-2 in addition to IL-15 in the SRG mice could further boost take rates and disease burden.

The in-depth molecular characterization of one of the SS PDX models generated in the SRG15 mice by scRNA-seq analysis relative to the original patient sample produced some interesting findings. Tracking of the clonal engraftment pattern by TCR sequencing demonstrated the presence of one dominant clone in the engrafted SRG15 mice, with a frequency ranging from 58% to 100% in skin and spleen. Curiously, this clone was different from the dominant clone in the patient sample. The strong selection for this TCR clone in the SRG15 mice suggests increased fitness of this particular clone in the murine microenvironment or increased responsiveness to the human IL-15. The study by Poglio et al. (2021) reported a complex clonal pattern in SS PDX and cell line models. Similarly, a discordant clonal engraftment pattern between primary tumor and PDX models has been documented in acute myeloid leukemia (Kawashima et al., 2022; Wang et al., 2017). Another interesting finding from the scRNA-seq analysis was the apparent differential regulation of gene expression in engrafted leukemic cells that homed to the skin versus the spleen, highlighting the impact of the tissue microenvironment. Although both skin- and spleen-resident Sezary cells represent the same clone, as assessed by TCR analysis, only the skin-resident Sezary cells exhibited the malignant Sezary cell gene expression signatures identified by Borcherding et al. (2019). It is currently unclear which host factors drive these tissue-specific differences in gene expression. Collectively, these data argue for the thorough molecular characterization of the established PDX models relative to the original patient samples to understand their translational impact.

Overall, we document an important function for IL-15 in CTCL and the utility of humanized IL-15 mice for the generation of SS PDX models. The SS PDX models generated in this study represent useful tools for the preclinical testing of therapeutic agents and complement an emerging set of recently developed preclinical SS platforms. Furthermore, these studies raise the possibility of a broader application of the SRG15 mice to the PDX model generation of additional T and NK cell malignancies and further validate the genetic humanization approach for the generation of mouse model of hematologic disease.

## MATERIALS AND METHODS

### Mice, patient samples and transplantation procedures

All mouse experiments were approved by Regeneron Pharmaceuticals. Recipient mice were 6- to 8-week-old female NOD-*scid* IL2Rgamma^null^ (NSG) mice purchased from The Jackson Laboratory, or human *SIRPA*^KI^*Rag2*^−/−^*Il2rg*^−/−^ (SRG) or human *IL15* knock-in on the SRG background (SRG15) (Herndler-Brandstetter et al., 2017) mice maintained in house. The care and use of experimental animals complied with all relevant institutional and national animal welfare laws, guidelines and policies. Frozen peripheral blood mononuclear cell (PBMC) samples of SS patient (Table S1) or control individuals were purchased from Discovery Life Sciences. Thawed PBMC cells (5×10^5^ to 1×10^6^) were intravenously transplanted into sublethally irradiated (2Gy) recipient mice. Blood samples were collected at time points indicated post transplantation. To prepare single-cell spleen suspensions from spleen, spleens were harvested, meshed through a 70 μM cell strainer, lysed for red blood cells, and resuspended in PBS with 2% fetal bovine serum. To prepare single-cell suspensions from skin, skin was minced into small pieces, softened in Hanks’ balanced salt solution at 37°C for 30 min, then digested with 10 mg/ml Collagenase D (Roche) at 37°C for 30 min twice. Cells were then separated by Ficoll gradient centrifugation. For later passages, 5×10^5^ to 1×10^6^ splenocytes or single cells prepared from skin were intravenously transplanted into sublethally irradiated (2Gy) recipient mice.

### *In vitro* proliferation assay

The human CTCL cell line Hut78 was purchased from American Type Culture Collection and cultured in RPMI with 10% fetal bovine serum. Short tandem repeat profiling was performed to confirm the cell line identity. For the proliferation assay, 500 cells were seeded in a 96-well plate, treated in technical triplicate with human cytokines purchased from R&D Systems or Peprotech for 3 days. The luminescence of viable cells was measured at different time points using CellTiter-Glo^®^ Luminescent Cell Viability Assay (Promega) according to the manufacturer's instructions.

### Quantitative PCR and western blotting

Hut78 cells were treated with JNK-IN-8 (Med Express) or IL-15 (R&D Systems) for the time indicated. RNA was extracted with a MagMAX Total RNA isolation kit (Invitrogen), reverse transcribed with a SuperScript III First-strand Synthesis system (Invitrogen). Quantitative PCR was performed in technical triplicate using SsoAdvanced Universal SYBR Green (Bio-Rad) and primers purchased from Integrated DNA Technology (Table S4). For western blotting, proteins were extracted in RIPA buffer with 1 mM phenylmethylsulfonyl fluoride (Cell Signaling Technology, 8553) and proteinase/phosphatase inhibitors (Cell Signaling Technology, 5872). Anti-c-Jun (Cell Signaling Technology, 9165; 1:1000), anti-phospho-c-Jun Ser63 (Cell Signaling Technology, 9261; 1:1000) and anti-tubulin (Cell Signaling Technology, 9099; 1:1000) antibodies were used for western blotting.

### Flow cytometry

Single-cell suspensions were prepared as described above. Antibody staining and fluorescence-activated cell sorting analysis were performed as previously described (Gao et al., 2009). The following monoclonal antibodies were used: anti-mouse CD45 (BioLegend, 30F11; 1:300), anti-mouse Ter119 (BioLegend, Ter119; 1:300), anti-human CD45 (BioLegend, HI30; 1:100), anti-human CD3 (BioLegend, OKT3; 1:100), anti-human CD2 (BioLegend, RPA-2.10; 1:100), anti-human CD4 (BioLegend, SK3; 1:100), anti-human CD5 (BioLegend, L17F12; 1:100), anti-human CD7 (BioLegend, CD7-6B7; 1:100), anti-human CD8 (BioLegend, RPA-T8; 1:100), anti-human ICOS (BioLegend, C398.4A; 1:100), anti-human PD-1 (eBioscience, MIH4; 1:5), anti-human CCR4 (eBioscience, 1G1; 1:100) and anti-human TRBC1 (BioLegend, JOVI.1; 1:100). Anti-human KIR3DL2 (FAB2878P) and anti-human EPHA4 (sc-365503 AF647) were purchased from R&D Systems and Santa Cruz Biotechnology, respectively, and used at 1:100. Antibodies were directly coupled to FITC, PE, PerCp5.5, PECy7, APC, APCCy7, Alexa Fluor 700, BV605 and BV711. Data were acquired on a BD Fortessa (BD Biosciences) and analyzed by FlowJo.

### Histology and immunohistochemistry

Paraffin blocks of skin samples from control or CTCL patients were purchased from Analytical Biological Services Inc. Immunohistochemistry was performed using anti-IL-15 (Abcam, ab55276, clone 3A3; 1:1000). Skin from PDX recipient mice was harvested, fixed in 10% formalin overnight and embedded in paraffin. Sections (4 μm) were cut and stained with Hematoxylin and Eosin (H&E), anti-human CD3 (Abcam, ab16669, clone SP7; 1:100) and 5 μg/ml anti-human PD-1 (Abcam, ab52587, clone NAT105; 1:200) at 4°C overnight.

### 5′ single-cell partitioning with gene expression and TCR library preparation, sequencing and read alignment

Single cells suspended in PBS with 0.04% bovine serum albumin were loaded, 10,000 cells per lane, on a Chromium Connect Single Cell Liquid Handler (10X Genomics). scRNA-seq and V(D)J libraries were prepared using a Chromium Next GEM Automated Single Cell 5′ Kit, v2 (10X Genomics). After amplification, cDNA was split into separate scRNA-seq and V(D)J aliquots. To enrich the V(D)J aliquot for TCR sequences, we used a Chromium Automated Single Cell Human TCR Amplification & Library Construction Kit (10X Genomics). Paired-end sequencing was performed on Illumina NovaSeq 6000 for RNA-seq libraries [read 128 bp for unique molecular identifier (UMI) and cell barcode, read 280 bp for transcript read, with 10 bp i7 and 10 bp i5 reads] and for V(D)J libraries (Read 1, 150 bp, 10 bp i7, 10 bp i5; Read 2, 150 bp). For RNA-seq libraries, Cell Ranger Single-Cell Software Suite (10X Genomics, v2.2.0) was used to perform sample demultiplexing, alignment, filtering and UMI counting. The human GRCh38 and mouse mm10 genome assembly and RefSeq gene model for human and mouse were used for the alignment. For V(D)J libraries, Cell Ranger Single-Cell Software Suite (10X Genomics, v2.2.0) was used to perform sample de-multiplexing, *de novo* assembly of read pairs into contigs, align and annotate contigs against all of the germline segment V(D)J reference sequences from human ImMunoGeneTics (IMTG), label and locate CDR3 regions, and group clonotypes.

### scRNA-seq and data analysis

mCD45^−^hCD45^+^hCD2^+^hCD4^+^hCD8^−^ cells were flow sorted from PBMCs of one unaffected donor and one SS patient, and single-cell suspensions prepared from skin and spleen from the four PDX mice described above. One spleen sample was dropped owing to low RNA. The total number of samples included in the single-cell analysis was 9. Single-cell analysis included quality control (QC), principal component analysis (PCA) and clustering using version 3 of the Seurat R package (Stuart et al., 2019). For QC, we removed cells with low gene counts, dead cells and doublets. These were filtered out as cells with fewer than 500 genes detected, cells with more than 20% mitochondrial reads and the top 3% of the UMI counts. UMI expression counts were log normalized with a UMI scaling factor of 10,000. For PCA, we found the top 2000 variable genes by computing the variability stabilizing transformation (vst) method to correct dependence of dispersion with a mean UMI between 0.0125 and 8 and dispersion above 0.5. The genes were then scaled and centered. The first 50 principal components (PCs) were used to run the UMAP dimensionality reduction. For the clustering analysis, all genes were projected into PC space (Seurat ProjectDim function), and 15 PCs were used as the number of dimensions to project onto (Seurat RunUMAP function). Clusters were then partitioned based on a 20 shared nearest neighbor graph (SNN) modularity optimization-based clustering algorithm (Seurat FindNeighbors and FindClusters functions). Resolution 0.3 resulted in 12 clusters of cells, which was sufficient for defining clusters. The two smallest clusters (10 and 11) were removed owing to their small sizes (62 and 24 cells, respectively). Differentially expressed genes were computed for each cluster compared to the rest of the clusters (Seurat FindAllMarkers function; significance was defined as *P*<0.01 and fold change >1.5).

Borcherding et al. (2019) identified malignant and normal CD4^+^ cells based on the top upregulated genes. We screened these genes against the differentially expressed genes in our clusters and defined them as malignant or normal CD4^+^ based on majority malignant genes or normal genes in each cluster. TCRs were identified using IgBLAST version 1.17.1 and aligned to the IMGT human genome (Ye et al., 2013). Unique TCRs were defined by V gene, J gene and CDR3 expression. Clone size, or clonotype expansion, was calculated for each sample and unique TCR as the number of cells with that TCR in each sample. Clone percentage was calculated as the clone size divided by the total number of TCRs per sample.

## Supplementary Material

10.1242/dmm.050190_sup1 Supplementary information
