# Supplementary material for: Microenvironment-dependent growth of Sezary cells in humanized IL-15 mice
Source: Dis Model Mech. 2023 Oct 13;16(10):dmm050190. doi: 10.1242/dmm.050190 (PMC10581384; doi:10.1242/dmm.050190)
Supplement: Supplementary information [file dmm-16-050190-s1.pdf]

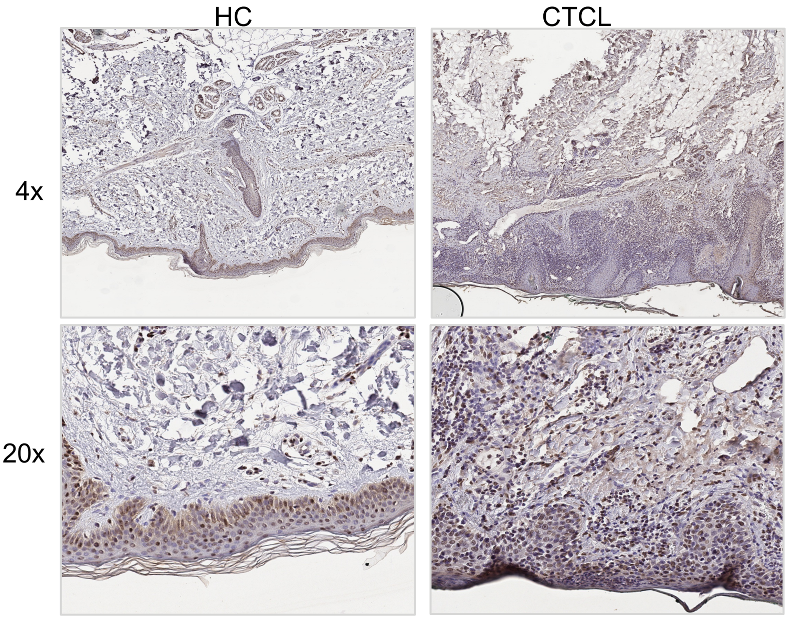

Fig. S1. Immunohistochemical analysis of IL-15 expression in skin biopsies from a healthy control (left) and CTCL patient (right).

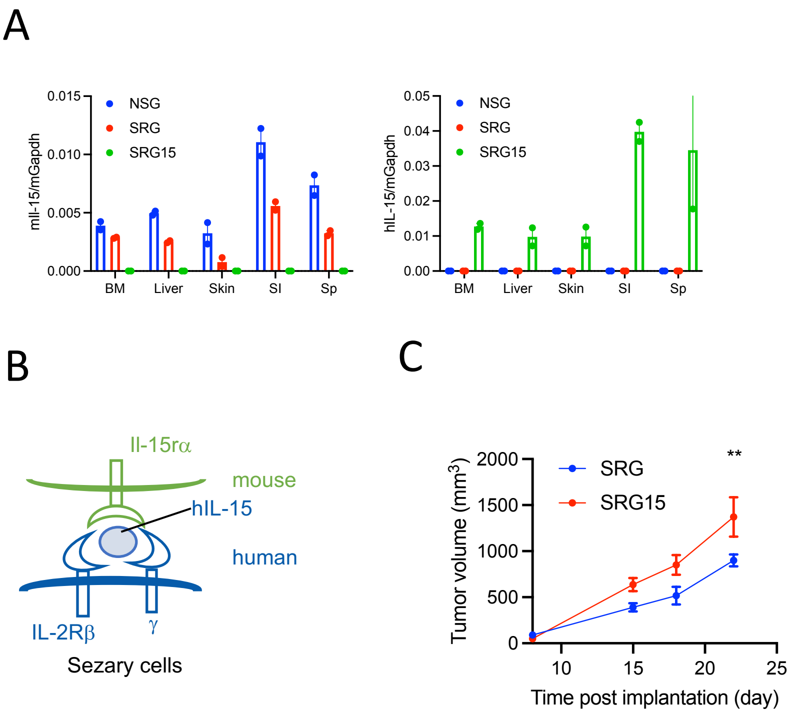

Fig. S2. Expression of IL-15 ligand and receptor components in SRG15 mice. (A) Taqman analysis of mouse and human IL-15 expression in the indicated tissues of NSG, SRG and SRG15 mice. BM, bone marrow; SI, small intestine; Sp, spleen. *Gapdh* was used as housekeeping gene control. Assays were set up in biological duplicate and technical triplicate. Data represent mean+/-SEM of biological duplicate. (B) Schematic representation of mouse and human IL-15 ligand and receptor interactions in SRG15 mice. (C) Hut78 tumor growth in SRG and SRG15 mice (n=5 per group). Data represent mean+/-SEM with 2way ANOVA Dunnett's multiple comparisons test at day 22. **p<0.01.

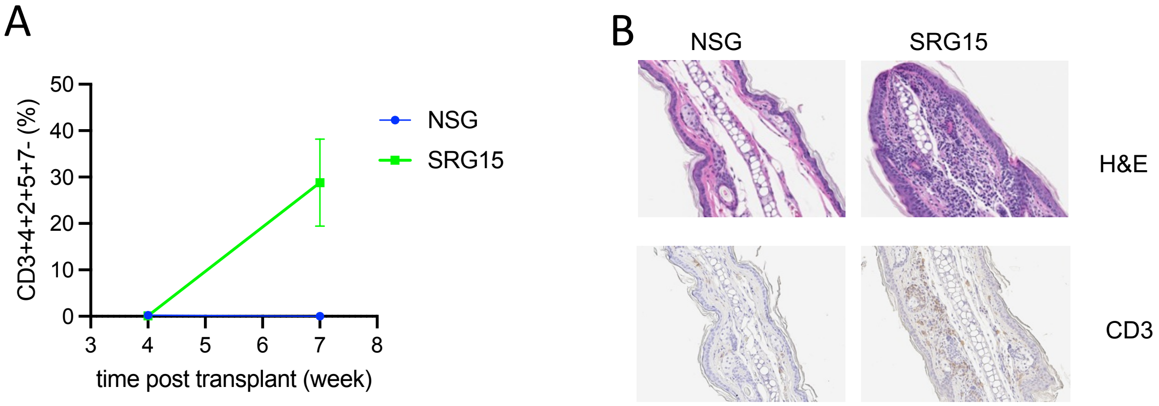

Fig. S3. SS PDX growth in SRG15 vs. NSG mice. (A) Patient sample 2 was xenografted into NSG and SRG15 mice (n=5 per group). Disease burden in peripheral blood as measured by frequency of $mCD45^{-}hCD45^{+}hCD3^{+}hCD4^{+}hCD2^{+}hCD7^{-}$ cells was plotted over time. Data represent mean +/-SEM. (B) H&E staining and CD3 IHC staining of ear punch specimens at 9-week post xenograft.

Disease Models & Mechanisms • Supplementary information

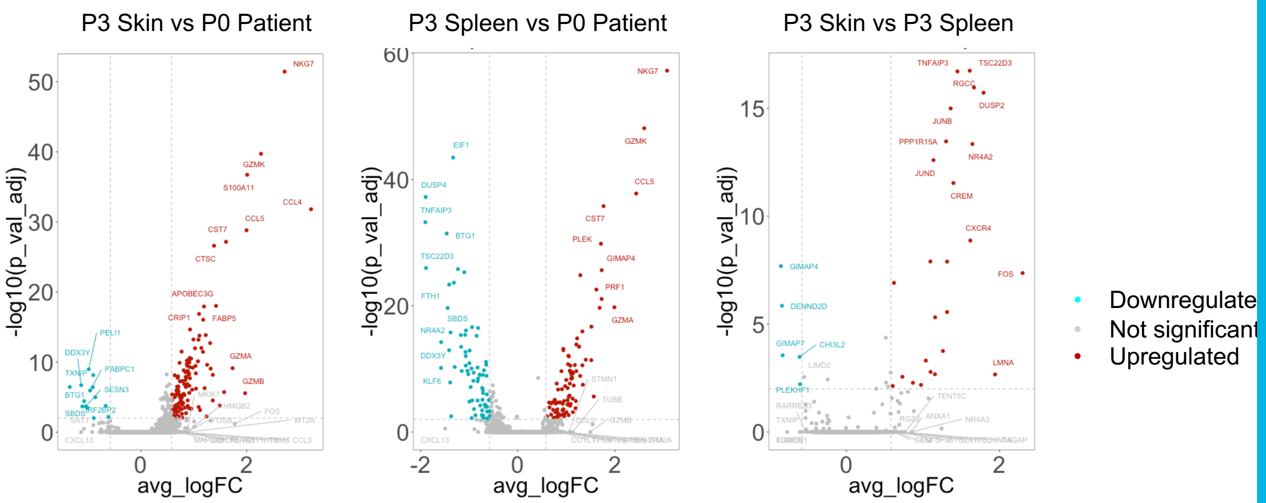

Fig. S4. Differentially expressed genes between Sezary cells of P0 patient, P3 spleen and P3 skin (related to Figure 4). The FindMarkers function was used in Seurat using the Deseq2 method for pseudobulk differential expression and a max of 50 cells per group. Genes were considered significant at fold change > 1.5 and adjusted p-value < 0.01.

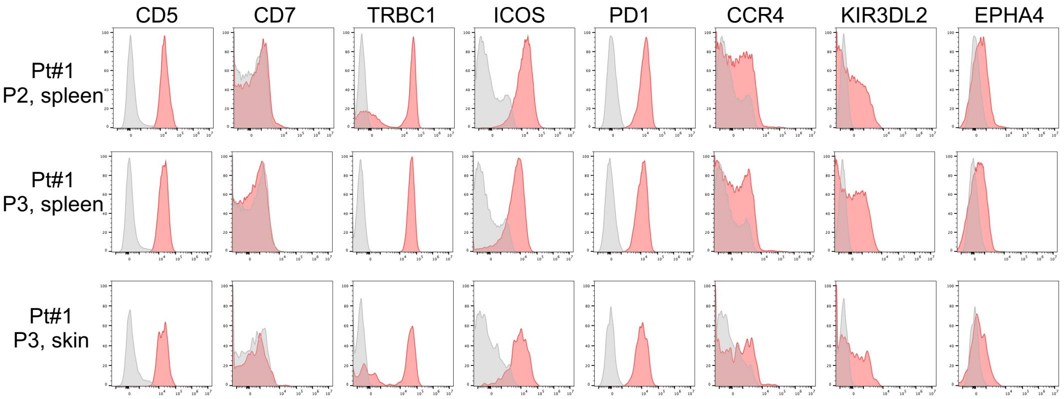

Fig. S5. Expression of cell surface markers on Sezary cells. Flow cytometry analysis of cell surface markers on Sezary PDX cells. Representative histogram of FMO control (grey line) and indicated genes (red lines) on Sezary cells (gated on hCD45^{+}mCD45^{-}CD4^{+}).

Disease Models & Mechanisms • Supplementary information

Table S1. SS patient characteristics

Patient ID	Primary Diagnosis	Gender	Race	Patient Age At Collection	Height (in)	Weight (lbs)	Tobacco History	Alcohol History	Treatment Status	Treatment Notes
1	Sezary syndrome (Stage IV-A)	Male	White	48	68	183	NA	No History	Pre Tx	NA
2	Sezary syndrome	Male	White	NA	70	188	Previous Use	Current Use	Active Tx	5th line of TX on Keytruda
3	Sezary syndrome	Female	Black	46	68	250	Never Used	No History	Active Tx	PUVA three times a week per visit
4	Sezary syndrome	Male	White	70	71	215	Previous Use	Current Use	Active Tx	Zolinza

Disease Models & Mechanisms • Supplementary information

Table S2. Pathway analysis of differentially expressed genes between Sezary cells of P0 patient, P3 spleen and P3 skin (related to Figure 4). GSEA enrichment analysis was used across Hallmark pathways using the Monte Carlo approach at 10,000 permutations. Pathways were considered significant at the p<0.01 level. Pathways with a minimum of 10 genes and a maximum of 600 genes were tested.

Comparison	Upregulated pathways	Downregulated pathways
P3 Skin vs. P0 Patient	Upregulated pathways in P3 Skin:<br>• HALLMARK_E2F_TARGETS<br>• HALLMARK_OXIDATIVE_PHOSPHORYLATION<br>• HALLMARK_MTORC1_SIGNALING<br>• HALLMARK_ALLOGRAFT_REJECTION<br>• HALLMARK_G2M_CHECKPOINT<br>• HALLMARK_MYC_TARGETS_V1<br>• HALLMARK_ESTROGEN_RESPONSE_LATE<br>• HALLMARK_INTERFERON_GAMMA_RESPONSE<br>• HALLMARK_APICAL_JUNCTION<br>• HALLMARK_COMPLEMENT	Downregulated pathways in P3 skin:<br>• HALLMARK_WNT_BETA_CATENIN_SIGNALING
P3 Spleen vs. P0 Patient	Upregulated pathways in P3 Spleen:<br>• HALLMARK_INTERFERON_ALPHA_RESPONSE<br>• HALLMARK_OXIDATIVE_PHOSPHORYLATION<br>• HALLMARK_E2F_TARGETS<br>• HALLMARK_APICAL_JUNCTION<br>• HALLMARK_INTERFERON_GAMMA_RESPONSE<br>• HALLMARK_ALLOGRAFT_REJECTION<br>• HALLMARK_COMPLEMENT	Downregulated pathways in P3 Spleen:<br>• HALLMARK_HYPOXIA<br>• HALLMARK_TNFA_SIGNALING_VIA_NFKB
P3 Skin vs. P3 Spleen	Upregulated pathways in P3 Skin:<br>• HALLMARK_TNFA_SIGNALING_VIA_NFKB<br>• HALLMARK_E2F_TARGETS<br>• HALLMARK_G2M_CHECKPOINT<br>• HALLMARK_HYPOXIA<br>• HALLMARK_EPITHELIAL_MESENCHYMAL_TRANSITION<br>• HALLMARK_MYC_TARGETS_V1<br>• HALLMARK_IL2_STAT5_SIGNALING<br>• HALLMARK_MYC_TARGETS_V2<br>• HALLMARK_APOPTOSIS<br>• HALLMARK_P53_PATHWAY<br>• HALLMARK_INFLAMMATORY_RESPONSE<br>• HALLMARK_UV_RESPONSE_UP<br>• HALLMARK_MTORC1_SIGNALING	Downregulated pathways in P3 skin:<br>• HALLMARK_ALLOGRAFT_REJECTION<br>• HALLMARK_APICAL_JUNCTION<br>• HALLMARK_INTERFERON_ALPHA_RESPONSE

Table S3. Overlapping gene signature from Borcherding et al. with the differentially expressed genes in each cluster. Percentage denominator for each cluster is the total number of overlapping genes. Clusters were defined as the majority overlap and ties were undetermined.

cluster	% Malignant CD4	% Normal CD4	Label	Malignant CD4 genes	Normal CD4 genes
cluster4	100	0	Malignant CD4	ID3, JUN, JUNB, NR4A2, SLC2A3, TTC39C, TTN, CCR7	
cluster2	91	9	Malignant CD4	CRIP1, S100A10, S100A11, S100A4, DUSP1, DUSP2, FOS, NR4A2, BIRC3, AHNAK	HLA-DQA1
cluster5	87.5	12.5	Malignant CD4	CRIP1, S100A10, S100A11, DUSP1, DUSP2, FOS, CDCA7	C12orf75
cluster6	33	67	Normal CD4	JUN, JUNB, CCR7	ABLIM1, C1orf162, SATB1, TXNIP, NUCB2, IL7R
cluster9	33	67	Normal CD4	CD70, CDCA7	NUCB2, PFN1, HLA-DRA, C12orf75
cluster1	25	75	Normal CD4	S100A4	CCL5, GZMA, NKG7
cluster7	25	75	Normal CD4	S100A6	TXNIP, IL7R, TIMP1
cluster8	20	80	Normal CD4	CDCA7	PFN1, HLA-DRA, HLA-DRB5, C12orf75
cluster0	12.5	87.5	Normal CD4	CD70	CCL5, HLA-DPA1, HLA-DPB1, HLA-DRB5, GZMA, GZMK, NKG7
cluster3	50	50	ND – not determined	DUSP1, JUN, JUNB, TTC39C	TXNIP, KLRB1, IL7R, TIMP1

Table S4. Primers for quantitative RT-PCR

Gene	Primer
IL2RB	IDT predesigned
IL2RG	IDT predesigned
IL4R	IDT predesigned
IL6R	IDT predesigned
IL7R	Forward: TCTCCCCGATCATAAGAAGA
	Reverse: TCTTGCAGAAAACCTTCCAC
IL9R	IDT predesigned
IL13RA1	IDT predesigned
IL13RA2	IDT predesigned
IL15RA	IDT predesigned
JUN	Forward: AAGGAAGCTGGAGAGAATCG
	Reverse: CAAAATGTTTGCAACTGCTG
JUNB	Forward: AATGGAACAGCCCTTCTACC
	Reverse: AAGAGGCGAGCTTGAGAGAC
GAPDH	Forward: GTCAGTGGTGGACCTGACCT
	Reverse: TGCTGTAGCCAAATTCGTTG
